# Supplementary material for: Association of Pre- and Gestational Conditions and Barriers to Breastfeeding with Exclusive Breastfeeding Practices
Source: Nutrients. 2025 Jul 13;17(14):2309. doi: 10.3390/nu17142309 (PMC12299373; doi:10.3390/nu17142309)
Supplement: Supplementary file 1 [file nutrients-17-02309-s001.zip › nutrients-3720570-supplementary.pdf]

Supplementary Table S1. Sociodemographic characteristics according to complete and incomplete cases, n=696.

Variables		Complete, *n*=566	Incomplete, n=130	**p*	$\chi 2$'s Pearson value
Age category					
	<20 y, n=79	63 (11.1)	16 (12.3)	0.621	0.951
	20-34 y, n=396	327 (57.8)	69 (53.1)		
	≥35 y, 221	176 (31.1)	45 (34.6)		
Occupation					
	Home, n=514	411 (72.6)	103 (79.2)	0.122	2.396
	Worker, 182	155 (27.4)	27 (20.8)		
Economic support					
	Herself, n=90	77 (13.6)	13 (10.0)	0.483	1.457
	Partner, n=461	370 (65.4)	91 (70.0)		
	Parents, n=145	119 (21.0)	26 (20.0)		
Civil status					
	Married, n=492	396(70.0)	96 (73.8)	0.380	0.769
	Single, n=204	170 (30)	34 (26.2)		
Socioeconomic level					
	Very low, n=226	186 (32.9)	40 (30.8)	0.871	0.276
	Low, n=308	248 (43.8)	60 (46.2)		
	Middle, n=162	132 (23.3)	30 (23.1)		
Educational level					
	Secondary and less, n=237	186 (32.9)	51 (39.2)	0.176	3.475
	High school, n=251	213 (37.6)	38 (29.2)		
	University, n=208	167 (29.5)	41 (31.5)		
Number of pregnancies					
	Multigravida, n=230	191 (33.7)	39 (32.0)	0.706	0.143
	Primigravida, n=458	375 (66.3)	83 (68.0)		

*p: p – value of Pearson's $\chi 2$

Supplementary Table S2. Type of risk and Breastfeeding Type, *n* (%).

Variable	Breastfeeding		
Type of risk	Exclusive, *n*=247	Non-exclusive, *n*=109	Formula, n=210
Non complicate, n= 80	46 (57.5)	13 (16.3)	21 (26.3)
Pregestational high risk, n=80	30 (37.5)	15 (18.8)	35 (43.8)
Gestational risk, n=174	85(48.9)	34 (19.5)	55 (31.6)
Pregestational and gestational high risk, n=232	86 (37.1)	47 (20.3)	99 (42.7)

$\chi 2$=14.952, p= 0.021
